# The Cytokine Response of Circulating Immune Cells to Rhinovirus (RV‐A16) is Reduced in Severe Asthma

**DOI:** 10.1111/jcmm.71410

**Published:** 2026-11-01

**Authors:** Kaschin Jamal Jameel, Sarah D. Yanik, Kai Robin Pfeifer, Daniel Weidinger, Simon Rohde, Birte Struck, Eike Bülthoff, Paul Bürger, Juliane Kronsbein, Matthias Tenbusch, Jürgen Knobloch

**Affiliations:** ^1^ Medical Clinic III for Pneumology, Allergology, and Sleep Medicine Bergmannsheil University Hospital, Ruhr University Bochum Bochum Germany; ^2^ Harald Zur Hausen Institute of Virology Uniklinikum Erlangen, Friedrich‐Alexander‐Universität (FAU) Erlangen‐Nürnberg Erlangen Germany; ^3^ FAU Profile Center Immunomedicine (FAU I‐MED) Friedrich‐Alexander‐Universität (FAU) Erlangen‐Nürnberg Erlangen Germany

**Keywords:** asthma, biomarker, exacerbations, human rhinovirus, infection, phenotypes

## Abstract

Severe asthma patients (SA) are more susceptible to respiratory infections with human rhinoviruses (HRV) than healthy subjects (H), causing exacerbations. In response to HRV, circulating immune cells are recruited to and activated at the local sites of infection. Hypothesis: The activation of circulating immune cells in response to HRV is impaired in severe asthma. We infected peripheral blood mononuclear cells (PBMC) of H (*n* = 20) and SA (*n* = 50) with RV‐A16 ex vivo. Cytokines were measured in culture supernatants by ELISA. Data were compared between SA and H and correlated to asthma phenotype biomarkers. RV‐A16 induced IFNα, IFNγ, CCL2, CCL5, TNFα, IL‐1β, IL‐6 and IL‐8. Except for CCL2, these cytokine responses were reduced in SA versus H. In SA, IFNα, IL‐6 and CCL5 responses correlated negatively, but IL‐1β correlated positively to FeNO. IFNγ correlated positively to blood eosinophils and serum IL‐33, and INFα correlated positively to serum total IgE. Cytokine responses of circulating immune cells to RV‐A16 are reduced in SA. Cytokines are differentially affected in allergic, eosinophilic or T2 low asthma. This could help to explain the increased risk for exacerbations in HRV infections in severe asthma and differences between phenotypes. Circulating immune cells might be considered as drug targets to reduce exacerbation rates.

## 1 Introduction

Asthma is a heterogeneous, multifactorial disease characterized by increased bronchial hyperreactivity, airflow variability and chronic airway and systemic inflammation [1, 2, 3, 4]. It affects 235 million people worldwide [5] of which about 5% suffer from severe disease stages [6]. Adequate diagnosis and appropriate treatment of these patients is a major challenge. Several phenotypes of severe asthma are characterized by frequent exacerbations that accelerate disease progression.

Viral respiratory infections are the leading cause of asthma exacerbations [7, 8]. In 83% of patients with asthma exacerbations, human rhinovirus (HRV) was detected [9]. Asthma patients have an increased susceptibility to viral and particularly to HRV infections [9, 10]. This implies defects in the immune response to HRV. The aim of our study was to find molecular pathologies and drug targets as a prerequisite to develop strategies to reduce infection‐induced exacerbations.

HRV induces innate and adaptive immune responses and affects local but also circulating immune cells [11, 12, 13]. In response to acute respiratory infections, peripheral blood mononuclear cells (PBMCs) are recruited to the draining lymph nodes and to the sites of infection [14, 15]. PBMCs include innate immune cells such as monocytes, dendritic cells and natural killer cells, as well as adaptive immune cells, like T cells and B cells [16]. The immune response to HRV includes the production of interferons, particularly IFNα and IFNγ, and of proinflammatory cytokines, such as TNFα, IL‐1β, IL‐6, IL‐8, CCL2 and CCL5 [12, 17, 18]. These cytokines are required for the control of HRV infection but can also promote airway inflammation in asthma [19, 20, 21, 22].

Asthma phenotypes are broadly regarded as T2‐high (type‐2 asthma) or T2‐low (non‐type‐2 asthma) [23]. T2‐high asthma is associated with type‐2 inflammation and can include T helper cells of the Th2 subtype, type‐2 innate lymphoid cells (ILC2), IgE‐secreting B cells and/or eosinophilic granulocytes [24]. Patients with severe allergic or eosinophilic asthma, both of which are T2‐high phenotypes, might benefit from monoclonal antibodies targeting IgE (omalizumab) or Type 2 cytokines and associated receptors. Such biologics particularly reduce exacerbations rates [4, 25]. However, while a variety of biologics have been approved for the different T2‐high asthma phenotypes in the last years, the possibilities for T2‐low asthma remain limited [25].

Biomarker‐based phenotyping has recently been coming into focus to identify putative responders for the different antibody treatments [3]. Total serum IgE is a suitable marker for identifying patients with allergic asthma that are candidates for therapy with omalizumab [23, 26]. Blood eosinophil levels are surrogate markers for sputum eosinophils. High levels indicate eosinophilic asthma and responsiveness to therapy with anti‐IL5 or anti IL‐5‐receptor antibodies [27, 28]. Fractionated exspiratory nitrogen monoxide (FeNO) indicates active Type 2 airway inflammation and correlates with blood eosinophil levels and exacerbation frequencies [29, 30, 31]. Serum periostin, TLSP and IL‐33 are biomarkers for asthma severity and/or phenotypes [32, 33].

Previous studies suggested hyperactivation of local immune cells and indicated a suppression of the activity of circulating immune cells in response to HRV in terms of cytokine production in mild‐to‐moderate asthma [8, 13, 34]. Thus, the infection defence might be impaired not only by defects in local but also in systemic immune cells [8]. However, although they could serve as potential drug targets to reduce exacerbations, the investigation of systemic defects in HRV‐associated immune responses in severe asthma has been neglected so far. We hypothesized that the activation of circulating immune cells in response to HRV is impaired in severe asthma and is influenced by type‐2 inflammation.

To address this question, we used human primary peripheral blood mononuclear cells (PBMC) as a culture model because they contain most of the circulating immune cells that are recruited to the draining lymph nodes or to the sites of infection in response to viral respiratory tract infections. We compared the cytokine response of PBMCs to RV‐A16 between severe asthma patients and non‐smoking healthy subjects. Furthermore, data were correlated with asthma biomarkers for inflammation and severity.

## 2 Material and Methods

### 2.1 Study Subjects

The study population consisted of 20 healthy never‐smokers (< 100 cigarettes in life) (H) and 50 severe asthma patients (SA) (Table 1) according to a sample size estimation (see below). Exclusion criteria for both groups were: < 18 years of age, pregnancy and reported infections within the last 2 weeks before sample preparation. Exclusion criteria for the H subjects were: FEV 1 < 80% pred. and any reported chronic disease. Inclusion criterion for the asthma group was a documented severe asthma according to criteria recommended by the National Institutes of Health and the Global Initiative for Asthma guidelines (GINA; Level 5). Exclusion criteria for the asthma group were current tobacco and e‐cigarette smoking (< 6 months smoking‐free), other lung diseases than asthma and any other systemic inflammatory or auto‐immune disease than asthma. 75 subjects were recruited. Five subjects were excluded because they did not meet the inclusion or exclusion criteria. Both groups were matched for age and sex (Table 1). The asthma patients were either naive to therapy with monoclonal antibodies or had not received monoclonal antibody treatment for at least 3 months before sample preparation. 11 SA subjects received systemic steroids (OCS) as continuous therapy at the time of sample preparation. Two subjects received 20 mg, three subjects received 10 mg, and 5 subjects received 5 mg OCS per day.

**TABLE 1:** Demographics of patients.

Parameter	H	SA
*N*	20	50
Gender (f:m)	13:7	28:22
Age (years)	55.35 ± 2.84	54.92 ± 1.47
FEV_1_ (% pred)	107.2 ± 3.59	74.93 ± 6.53****
FEV1/FVC (%)	82.2 ± 1.76	69.75 ± 1.69***
Monocytes (% wbc)	4.26 ± 0.49	7.74 ± 0.31****
Lymphocytes (% wbc)	37.32 ± 2.79	26 ± 1.09***
Neutrophils (% wbc)	56.35 ± 2.9	60.79 ± 1.46
Eosinophils (% wbc)	1.84 ± 0.38	4.75 ± 0.57***
Eosinophils (cells/μL)	n.d.	369.8 ± 55.95 (*n* = 48)
Serum total IgE (IE/mL)	n.d.	923.2 ± 434.2 (*n* = 48)
Serum Periostin (ng/mL)	n.d.	65.6 ± 10.24 (*n* = 48)
Serum TSLP (pg/mL)	n.d.	762.8 ± 245.1 (*n* = 47)
Serum IL‐33 (pg/mL)	n.d.	8.32 ± 0.93 (*n* = 48)
FeNO (ppm)	n.d.	51.47 ± 9.9 (*n* = 38)

*Note:* Asthma patients were GINA Stage V (Global Initiative for Asthma). Data have been collected at the day of sample preparation and are presented as mean ± standard error of the mean (SEM).

Abbreviations: FEV1, forced expiratory volume in 1 s; H, healthy subjects; SA, severe asthma patients; WBC, white blood cells.

*** *p* < 0.001.

**** *p* < 0.0001.

The primary endpoint of our study was the comparison in cytokine release in response to RV‐A16 between healthy and severe asthma subjects. According to our hypothesis, the sample size estimation was based on the data of Bedke et al. [35]. For the deviation a *σ*1 = 3.3 as well as a *σ*2 = 3, and for the true difference of mean a *δ* = 4.3 was determined. With an *α* < 0.05 and a power (1‐β) of 0.9 the included number of subjects was estimated to be *n* = 13 per group. We increased the numbers to *n* = 20 for H and *n* = 50 for SA to compensate for potential dropouts and to analyse cytokine production in correlation to asthma biomarkers as secondary endpoints. To this end, total serum IgE (*n* = 48), blood eosinophils (*n* = 48), FeNO (*n* = 38), serum periostin (*n* = 48), serum TSLP (*n* = 47), or serum IL‐33 (*n* = 48) data were used from asthma subjects, if available (Table 1). The study has been approved by the Ethics Committee of the Ruhr University Bochum, Bochum, Germany (ID: 4257‐12, 4772‐13).

### 2.2 Isolation of Peripheral Blood Mononuclear Cells (PBMCs) and Infection With Human Rhinovirus (RV‐A16 Strain)

The isolation of PBMCs via Ficoll gradient sedimentation, cell counts and vitality tests via trypan blue staining were performed according to established protocols [36]. 0.5 × 10^6^ PBMCs were cultured in 500 μL RPMI 1640 medium (Sigma, Munich, Germany) with 2 mM L‐glutamine, 100 U/mL penicillin and 100 μg/mL streptomycin (all from Sigma) per approach. Cells were directly infected with RV‐A16 (Virapur, Lot# G1118A) at a multiplicity of infection (MOI) 1 or 0.1. PBMCs were cultivated after infection for 16 h at 5% CO_2_ and 37°C without FCS. Then, 10% FCS was added to the culture medium. Two time points were chosen for the cytokine response analyses: 24 h after infection, addressing primarily responses of innate immune cells, and 7 days after infection, which additionally considers putative responses of T cells. To this end, cytokine concentrations were analysed 24 h or 7 days after infection in the supernatants. For some experiments, RV‐A16 was inactivated by exposition to 254 nM UV light for 45 min before adding to the cells.

### 2.3 RNA Isolation and cDNA Synthesis

RNA isolation from supernatants was performed with the NucleoSpin RNA Virus KIT (Machery Nagel) according to the manufacturer's instructions with the use of DNaseI. RNA concentrations were determined using a NanoDrop1000 (Nanodrop) according to the manufacturer's instructions. Samples were stored at −80°C. cDNA synthesis was performed with an Omniscript RT KIT (Qiagen) according to the manufacturer's instructions. The cDNA was stored at −80°C.

### 2.4 Rotorgene Reverse Transcription Quantitative PCR (RT‐qPCR)

For the quantification of RV‐A16 in supernatants, the RNA was isolated at days 1 and 7 and subjected to RT‐qPCR with the QuantiTect KIT (Qiagen) according to the instructions of the manufacturer with SYBRGreen. Primer sequences were 5′‐GTGAAGAGCCSCRTGTGCT‐3′; 5′‐GCTSCAGGGTTAAGGTTAGCC‐3′.

### 2.5 Enzyme‐Linked Immunosorbent Assay

IFNα, IL‐1β, TNFα, IL‐6, IL‐8, IFNγ, CCL5 and CCL2 concentrations in the supernatants of cultured PBMCs were measured by ELISA according to the manufacturer's instructions (Invitrogen, Waltham, Massachusetts; cat# BMS216MST, cat# 88‐7261‐88, cat# 88‐7346‐88, cat# 88‐7066‐88, cat# 88‐8086‐88, cat# 88‐7316‐88; and R&D Systems, Minneapolis, MN; cat# DY278) and as described in [37, 38].

### 2.6 Statistical Analysis

The Wilcoxon signed‐rank test was used to analyse differences between approaches within a group. For comparisons between H and SA, a two‐tailed Mann–Whitney test was used. Spearman's rank correlation test was performed to correlate cytokine data with biomarkers. A *p* < 0.05 was considered statistically significant. All tests were performed using GraphPad Prism 7 (Graph Pad Software; San Diego, CA, USA).

## 3 Results

### 3.1 Differences Between Study Groups

As expected, SA subjects had reduced lung function parameters compared to H (Table 1). SA subjects had also higher ratios of monocytes and eosinophils but lower ratios of lymphocytes on white blood cells than H (Table 1). Gender ratio and age were not different between the groups (Table 1). All study subjects did not report symptoms of acute infections in the last 14 days before sample preparation or on systemic inflammatory or autoimmune diseases (with the exception of asthma in the SA group). Therefore, the PBMCs should not have been recently activated before isolation. At the start of the cultivation, the PBMCs of the H group should not have an active status at all whereas those of the SA group might mirror a putative asthma‐caused baseline inflammatory status.

### 3.2 Baseline Cytokine Levels of PBMCs Are Lower in Severe Asthma (SA) Versus Healthy Subjects (H)

PBMCs of the study subjects were cultured with or without the addition of RV‐A16 at MOI 0.1 or 1.0. to the culture medium for 24 h or 7 days. Baseline release (cultivation in the absence of RV‐A16) of TNFα, IL‐6, IL‐8 and IFNγ were lower in PBMCs of SA versus H after 24 h and/or 7 days of cultivation (Figure 1C–E,H). IL‐1β was increased in SA (Figure 1B), and baseline levels of IFNα, CCL2 and CCL5 were not significantly different between these two groups (Figure 1A,F,G).

**FIGURE 1:**
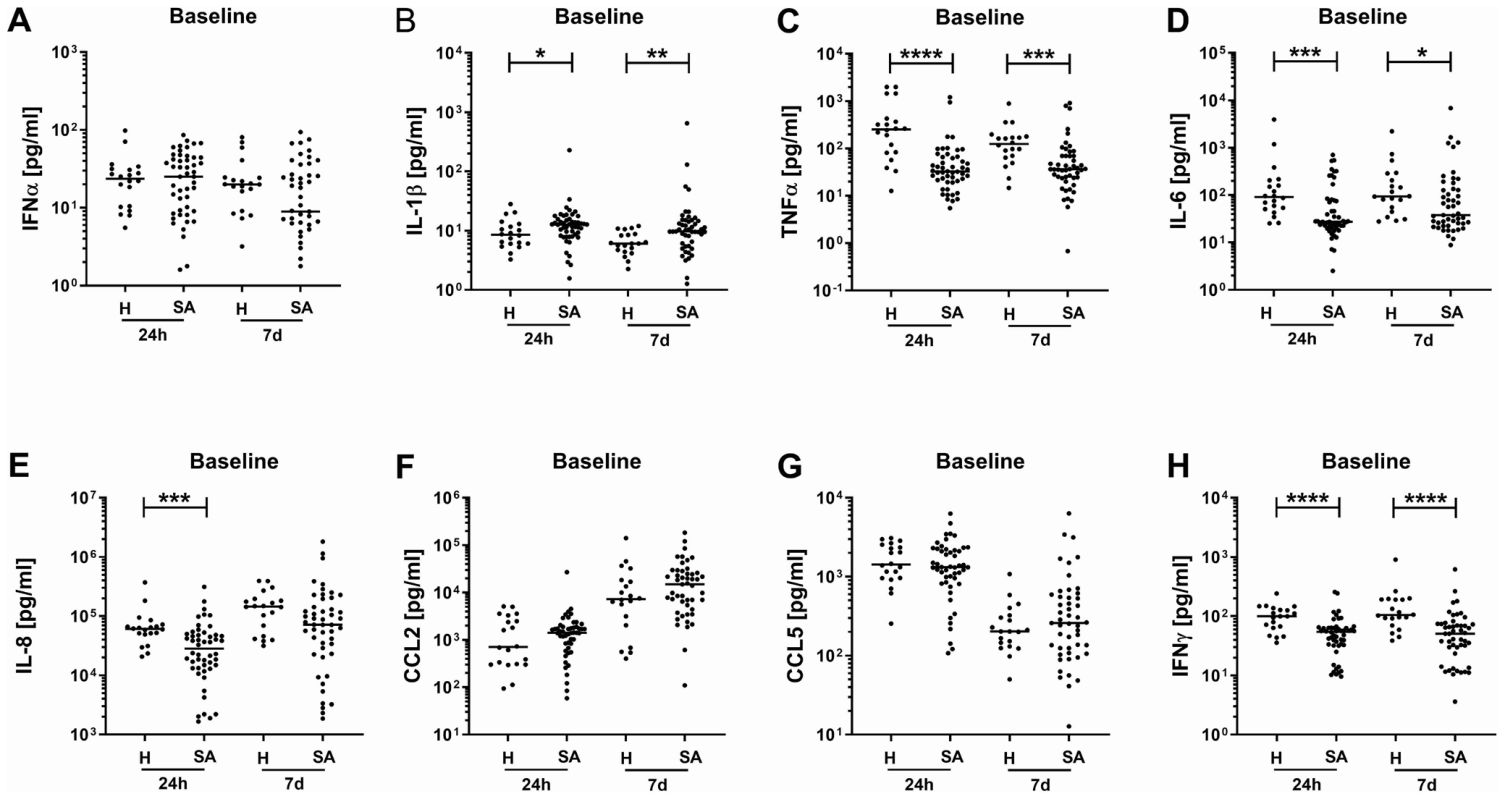
Baseline release of cytokines from PBMCs of severe asthma patients compared to healthy subjects. PBMCs from 20 healthy (H) or 50 asthma subjects (SA) (for 7 days, *n* = 49) were cultivated for 24 h or 7 days. IFNα (A), IL‐1β (B), TNFα (C), IL‐6 (D), IL‐8 (E), CCL2 (F), CCL5 (G), and IFNγ (H) were measured in the supernatants by ELISA. Data are presented as median with scatter. Data were compared between the two groups with the Mann–Whitney test: *p < 0.05, **p < 0.01, ***p < 0.001, ****p < 0.0001.

### 3.3 Cytokine Production of PBMCs in Response to RV‐A16 Is Reduced in Severe Asthma

The effects of RV‐A16 on the cytokine response of PBMCs after 24 h and 7 days were analysed on the absolute amounts of cytokine concentrations in the culture supernatants (Table 2) and after subtraction of baseline data (non‐infected reference cell cultures, refer to Figure 1; Table 3). RV‐A16 induced IFNα, IL‐1β, TNFα, IL‐6, IL‐8, CCL2, CCL5 and IFNγ in H in a dose‐ and time‐dependent manner (Tables 2 and 3). RV‐A16 also induced these cytokines, with the exception of IL‐1β, in SA (Tables 2 and 3). Except for CCL2, all these responses were reduced in SA compared to H depending on RV‐A16 dose and incubation time (Tables 2 and 3). In contrast to the 24 h incubation time, TNFα was suppressed by RV‐A16 at MOI 1.0 after 7 days in both groups without statistically significant differences (Table 3). RV‐A16 did not induce GM‐CSF, IL‐4, IL‐5, IL‐13, or IL‐17 at these conditions in PBMCs (data not shown).

**TABLE 2:** Interferon and cytokine levels in cultured PBMCs infected with RV‐A16.

Cytokine	MOI	Time	Concentration [pg/mL]; median, interquartile range			
			Healthy		Severe asthma	
IFNα	0.1	24 h	25.29	18.28; 66.70	16.56	7.163; 45.61^#^
		7 days	25.47	17.04; 88.91^##^	18.63	7.500; 39.44*
	1.0	24 h	102.2	36.50; 268.8^####^	28.06	8.904; 68.72***
		7 days	105.5	33.41; 247.9^####^	24.86	7.790; 61.87^#^***
IL‐1β	0.1	24 h	15.80	5.555; 175.4^##^	11.24	6.913; 14.97
		7 days	7.950	4.673; 256.3^###^	10.68	7.005; 15.41
	1.0	24 h	12.03	5.278; 416.4	13.58	9.240; 18.74
		7 days	8.725	2.850; 295.1^##^	9.670	6.880; 13.62
TNFα	0.1	24 h	510.3	121.1; 2605^##^	28.28	16.15; 77.43****
		7 days	114.1	41.52; 208.0	37.75	24.32; 81.15***
	1.0	24 h	1102	290.5; 2026^##^	36.02	23.53; 84.99****
		7 days	73.06	41.72; 100.2^##^	30.80	19.03; 41.28^#^***
IL −6	0.1	24 h	244.4	93.53; 2909^###^	41.46	21.91; 175.6^##^****
		7 days	455.2	129.8; 3303^####^	95.53	56.14; 317.5^####^**
	1.0	24 h	841.1	213.1; 3958^####^	41.72	25.58; 4296^####^****
		7 days	1706	371.0; 2839^####^	196.0	54.56; 520.7^####^****
IL‐8	0.1	24 h	69,223	38,451; 263,506	31,970	14,797; 55,039***
		7 days	323,387	90,936; 676,792^##^	79,389	43,648; 199,307**
	1.0	24 h	68,141	34,042; 208,466	41,150	20,585; 61,037^#^**
		7 days	192,166	66,421; 344,903	99,439	26,616; 158,343^#^*
CCL2	0.1	24 h	1531	295.7; 7042	1965	784.1; 3327^##^
		7 days	25,786	5732; 47,855^###^	22,347	9719; 56,385^###^
	1.0	24 h	2492	378.5; 6075^##^	2888	1936; 5130^####^
		7 days	6404	997.3; 72,897^#^	40,621	14,205; 78,411^####^
CCL5	0.1	24 h	2364	962.0; 3241	1242	852.4; 1831*
		7 days	291.7	211.7; 742.2^#^	264.3	119.3; 846.6
	1.0	24 h	2441	1330; 3570^###^	1431	804.2; 2173**
		7 days	580.1	260.5; 1741^####^	293.9	156.3; 839.9^##^
IFNγ	0.1	24 h	92.08	48.98; 124.1	53.59	39.22; 72.02^#^
		7 days	253.0	126.5; 6713^###^	62.69	48.36; 146.5^####^****
	1.0	24 h	141.1	87.06; 181.0	55.91	39.95; 94.44^##^***
		7 days	503.5	201.9; 1953^####^	106.6	54.41; 288.8^####^***

*Note:* PBMCs from 20 healthy (H) or 50 asthma subjects (SA) (for 7 days, *n* = 49) were stimulated with RV‐A16 at MOI 0.1 and 1 for 24 h or 7 days. IFNα, IL‐1β, TNFα, IL‐6, IL‐8, CCL2, CCL5, and IFNγ were measured in the supernatants by ELISA. Differences to the baseline (Figure 1) were analysed using the Wilcoxon test: ^#^ *p* < 0.05, ^##^ *p* < 0.01, ^###^ *p* < 0.001 and ^####^ *p* < 0.0001. Differences between H and SA were analysed by the Mann–Whitney test: test: *p < 0.05, **p < 0.01, ***p < 0.001 and ****p < 0.0001.

**TABLE 3:** RV‐A16‐induced interferon and cytokine secretion in PBMCs.

Cytokine	MOI	Time	△ concentration (induction minus baseline) [pg/mL]: median; interquartile range			
			Healthy (H)		Severe asthma (SA)	
IFNα	0.1	24 h	0.01000	−5.168; 33.47	−2.415	−11.54; 1.638
		7 days	4.570	1.560; 27.60^##^	1.275	−2.105; 9.348
	1.0	24 h	76.42	12.49; 240.1^####^	0.1500	−4.488; 24.60***
		7 days	87.86	16.25; 223.1^####^	3.960	−3.103; 30.95^#^***
IL‐1β	0.1	24 h	2.650	−0.4125; 164.9^##^	−1.160	−3.595; 1.053***
		7 days	2.065	0.4175; 246.7^###^	−0.07000	−1.928; 2.208**
	1.0	24 h	3.720	−1.358; 405.9	0.6150	−3.280; 4.640
		7 days	2.105	−0.04750; 285.8^##^	−0.2450	−2.493; 2.798**
TNFα	0.1	24 h	200.8	−0.6275; 1162^##^	−2.185	−11.14; 19.74**
		7 days	−11.07	−40.97; 20.66	0.1150	−11.00; 16.73
	1.0	24 h	236.8	4.348; 1207^##^	4.355	−8.985; 24.04***
		7 days	−20.03	−75.61; −1.908^##^	−5.810	−18.76; 2.340^#^
IL‐6	0.1	24 h	87.71	8.038; 2391^###^	7.875	−2.755; 47.39^##^**
		7 days	257.8	30.15; 2744^####^	32.72	3.935; 106.1^####^**
	1.0	24 h	713.5	150.2; 2467^####^	10.27	2.128; 3590^####^****
		7 days	916.4	142.4; 2795^####^	61.75	9.290; 340.9^####^***
IL‐8	0.1	24 h	6597	−10,530; 71,052	708.2	−3277; 7909
		7 days	91,820	−2409; 522,200^##^	242.2	−9844; 38,803*
	1.0	24 h	8517	−16,012; 123,549	3130	−2220; 12,499^#^
		7 days	30,909	−17,452; 156,646	4934	−5281; 41,379^#^
CCL2	0.1	24 h	58.24	−33.10; 4222	207.0	−27.25; 1084^##^
		7 days	16,962	212.7; 31,255^###^	3250	−739.1; 24,448^###^
	1.0	24 h	1347	44.10; 4287^##^	1642	523.7; 3715^####^
		7 days	1957	−343.8; 61,974^#^	17,369	510.8; 56,869^####^
CCL5	0.1	24 h	106.8	−68.76; 920.4	−72.88	−296.6; 196.2*
		7 days	48.12	−9.545; 618.4^#^	9.760	−42.37; 124.6
	1.0	24 h	488.0	87.52; 1029^###^	27.37	−213.3; 206.3***
		7 days	362.0	74.51; 902.8^####^	55.89	−20.86; 196.9^##^**
IFNγ	0.1	24 h	−5.365	−20.06; 1.905	2.210	−6.595; 39.03
		7 days	106.5	7.338; 6622^###^	29.07	0.2625; 78.95^####^*
	1.0	24 h	6.405	−14.90; 95.42	21.08	−11.68; 40.20^##^
		7 days	346.5	51.78; 1740^####^	69.10	22.26; 230.0^#####^*

*Note:* PBMCs from 20 healthy (H) or 50 asthma subjects (SA) (for 7 days, *n* = 49) were stimulated with RV‐A16 at MOI 0.1 and 1 for 24 h or 7 days. IFNα, IL‐1β, TNFα, IL‐6, IL‐8, CCL2, CCL5, and IFNγ were measured in the supernatants by ELISA. Data were subtracted from the baseline (� concentration). Differences to the baseline (hypothetical value = 0) were analysed using the Wilcoxon test: ^#^ *p* < 0.05, ^##^ *p* < 0.01, ^###^ *p* < 0.001 and ^####^ *p* < 0.0001. Differences between H and SA were analysed by the Mann–Whitney test: test: *p < 0.05, **p < 0.01, ***p < 0.001 and ****p < 0.0001.

### 3.4 RV‐A16‐Induced Cytokine Response in Correlation to Asthma Biomarkers

Next, we correlated the cytokine response data after baseline subtraction to the asthma biomarkers serum total IgE, blood eosinophils, FeNO and serum periostin, TSLP and IL‐33. Only those cytokine data were considered that showed a statistically significant difference between the SA and the H group in Table 3. We found that the IFNα response to RV‐A16 positively correlated to total IgE but negatively to FeNO (Table 4). The IFNγ response after 7 d positively correlated to eosinophils and IL‐33 (Table 4). IL‐1β responses after 7 days positively correlated but the CCL5 and IL‐6 responses after 24 h negatively correlated to FeNO (Table 4). No correlations were found of any cytokine response to serum periostin or TSLP (data not shown).

**TABLE 4:** Correlations of RV‐A16‐induced cytokine responses to asthma biomarkers.

Biomarker	Cytokine (RV‐A16 concentration; infection time)	*r*_s_ spearman	*p*
Total IgE (IE/mL)	IFNα (MOI 1; 24 h)	0.3395	0.0182
Eosinopils (cells/μl)	IFNγ (MOI 1; 7 days)	0.4310	0.0025
FeNO (ppb)	IFNα (MOI 1; 24 h)	−0.4070	0.0112
	IL1β (MOI 0.1; 7 days)	0.3720	0.0234
	IL1β (MOI 1; 7 days)	0.4236	0.0090
	CCL5 (MOI 1; 24 h)	−0.4560	0.0040
	IL‐6 (MOI 0.1; 24 h)	−0.3638	0.0248
Serum IL‐33 (pg/mL)	IFNγ (MOI 0.1; 7 days)	0.3613	0.0126

*Note:* Data from Table 3 (cytokine response to RV‐A16 subtracted from baseline) were correlated with the biomarkers serum total IgE (24 h/7 days: *n* = 48/47), blood eosinophils (*n* = 48/47), FeNO (*n* = 38/37) and serum periostin (*n* = 48/47), TSLP (*n* = 47/46), and IL‐33 (*n* = 48/47) using Spearman's rank correlation test.

Abbreviation: *r*_s_ = spearman's rank correlation coefficient.

### 3.5 RV‐A16 Replication in PBMCs Induces Cytokine Production

Finally, we investigated whether RV‐A16 replicates in PBMCs and if this is a prerequisite for the induction of the expression of the investigated cytokines in healthy subjects. RV‐A16 copy numbers above the input were detected in supernatants of PBMC cultures after 24 h and 7 days of incubation with replication‐competent RV‐A16 but not after incubation with UV‐inactivated RV‐A16 (Figure 2) confirming productive replication of RV‐A16 in PBMCs. Next, we analysed the cytokine concentrations in the supernatants. Replication‐competent RV‐A16 induced all cytokines except CCL2 stronger than UV‐inactivated RV‐A16 (Figure 3). We conclude that cytokine release of PBMCs in response to RV‐A16 primarily depends on active replication. However, CCL2 secretion is also triggered by inactivated particles, suggesting direct recognition of viral particles or sensing of incoming vRNA after endocytosis.

**FIGURE 2:**
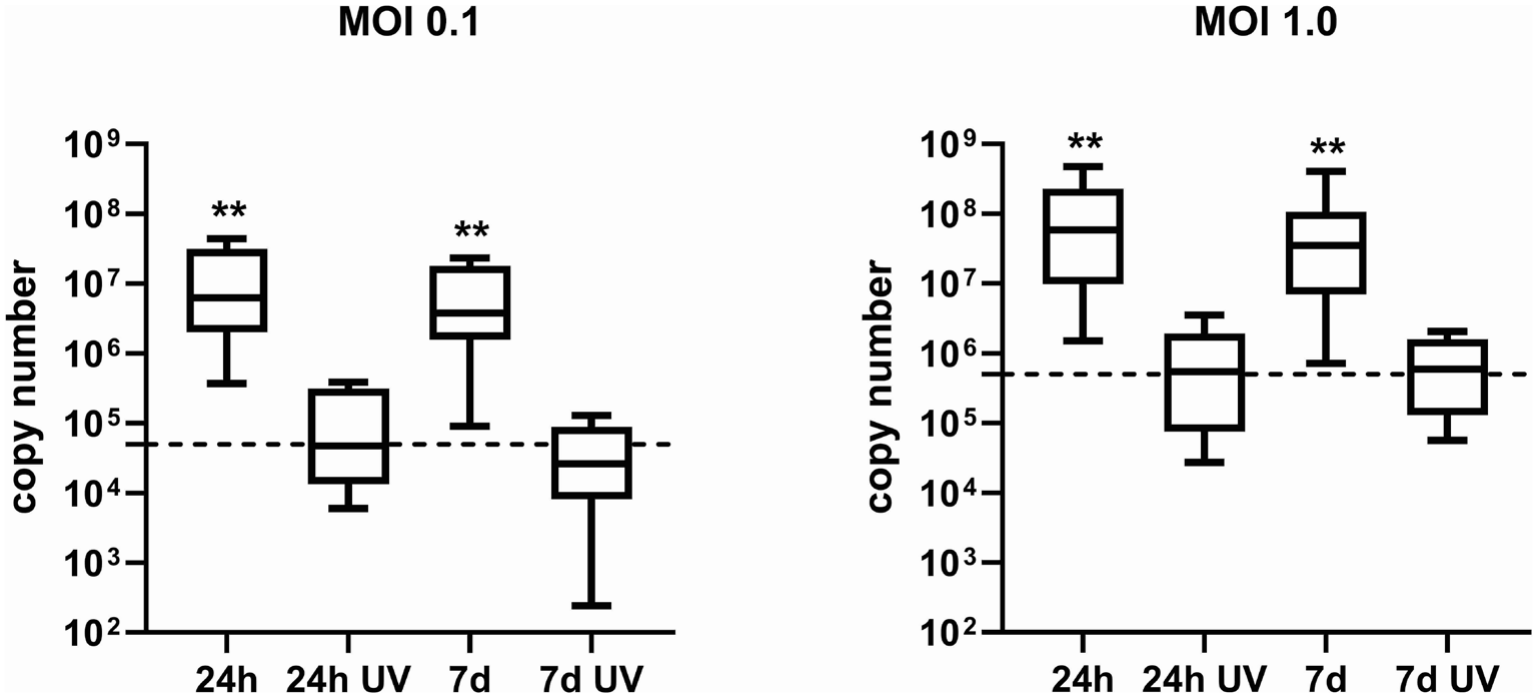
RV‐A16 replicates in PBMCs. PBMCs from eight healthy subjects were incubated with replication‐competent RV‐A16 or with UV‐inactivated RV‐A16 at MOI 0.1 and 1 for 24 h or 7 days. RV‐A16 copy numbers were detected by real time PCR on culture supernatants. Data are presented as a 10–90 percentile box‐and‐whisker plot. Data were compared with a Wilcoxon signed rank test versus the input: **p < 0.01. Dashed line: HRV input.

**FIGURE 3:**
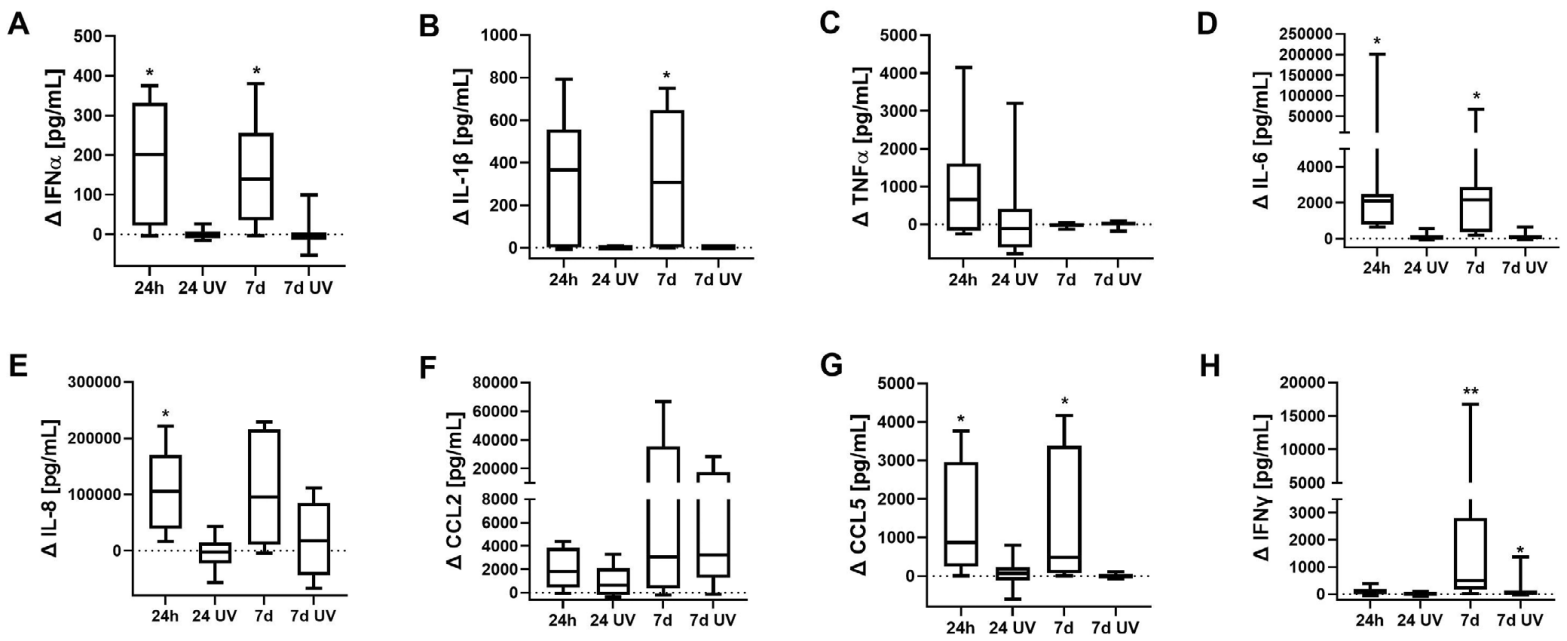
RV‐A16 replication induced cytokine release of PBMCs. PBMCs from five to eight healthy subjects were incubated with replication‐competent or UV‐inactivated RV‐A16 at MOI 1 for 24 h or 7 days. IFNα (A), IL‐1β (B), TNFα (C), IL‐6 (D), IL‐8 (E), CCL2 (F), CCL5 (G), and IFNγ (H) were measured in the supernatants by ELISA. Data were subtracted from the baseline (△ concentration) are presented as a 10‐90 percentile box‐and‐whisker plot. Differences to the baseline (hypothetical value = 0) were analysed using the Wilcoxon test: *p < 0.05, **p < 0.01. 24h, 7d: incubation with replication competent RV‐A16 for 24h or 7d; 24 UV, 7d UV: incubation with UV‐inactivated RV‐A16 for 24h or 7d.

## 4 Discussion

Exacerbations induced by viral infections of the respiratory tract, for example, with HRV, are an important trigger for asthma progression because they increase local and systemic inflammation [8, 39, 40]. Infection of local cells such as airway epithelial cells and macrophages with human rhinovirus causes the release of interferons and cytokines [34]. These inflammatory mediators trigger the recruitment of antigen‐presenting cells (APCs) from the circulation, such as monocytes and DCs [19, 20, 21, 22]. APCs activate circulating adaptive immune cells, T and B lymphocytes, in the draining lymph nodes from which they are recruited to the sites of infection and contact the pathogens [8, 34]. Thus, circulating immune cells are critically involved in viral clearance, and ex vivo infection of PBMCs is a simple cell culture model to study the response of circulating immune cells to human rhinovirus.

Asthma patients are more susceptible to HRV infections than healthy subjects [9, 10]. Therefore, we hypothesized that the immune response to HRV is impaired in severe asthma patients. The underlying reasons for the increased susceptibility of asthma patients to HRV infections have been poorly investigated on the cellular level so far, although this is a prerequisite for developing drug target strategies [22, 41]. We hypothesized that systemic defects in circulating cells prevent their full activation in response to HRV in severe asthma. In support of this hypothesis, we have previously found a reduced response of circulating T‐cells and/or monocytes to lipopolysaccharide from gram‐negative bacteria, to non‐typeable *Haemophilus influenzae* , or to gram‐positive PAMPs in mild‐to‐moderate asthma or chronic obstructive pulmonary disease, respectively [38, 42, 43, 44]. To investigate the hypothesis, we infected PBMCs ex vivo with RV‐A16 and analysed endpoint activation markers in terms of cytokine release. We used PBMCs because this cell culture model includes almost all relevant circulating immune cells, monocytes, dendritic cells, natural killer cells, T cells and B cells [16] and considers their interactions.

Baseline levels of TNFα, IL‐6, IL‐8 and IFNγ from PBMCs were reduced in SA compared to H. Previous studies have shown increased TNFα, IL‐6 and IL‐8 levels in the serum of severe asthma patients [45, 46, 47]. These increased cytokine levels have been associated with increased numbers of circulating neutrophils [47], which are not present in PBMC cultures. TNFα and IL‐8 are particularly produced by monocytes [43], which were increased in the SA group. Therefore, our data indicate that circulating mononuclear cells might not contribute to the systemic increase of these cytokines in severe asthma and that an increased neutrophilic activity might overcome the reduced TNFα, IL‐6 and IL‐8 baseline production of PBMCs. High cytokine levels in serum are associated with systemic inflammation and poor asthma control [45, 46, 47]. However, for IFNγ, one study suggests that a lower serum level of IFNγ may be associated with an increased severity of airway obstruction [48] and our data indicate that the reduced IFNγ release of PBMCs might contribute to this. IFNγ production from unstimulated PBMCs might mainly come from natural killer cells because non‐activated T‐cells do not produce sufficient amounts to be detected with the ELISA method used in this study. Thus, the reduced lymphocyte counts in SA might not be the reason for the reduced baseline IFNγ. Baseline IL‐1β was increased in SA compared to H, whereas IFNα, CCL2 and CCL5 were not different between the two groups. IL‐1β is a key mediator in T2 low neutrophilic asthma phenotypes [49]. Elevated IL‐1β levels appear to play a role in virus‐induced asthma exacerbations [50] and our data indicate that PBMCs might contribute to this.

HRV has a significant impact on asthma exacerbations and is associated with more severe clinical conditions [5, 51]. Our data showed reduced interferon and cytokine releases from PBMCs in response to RV‐A16 in SA compared to H. This suggests a reduced activity of immune cells that are recruited from the circulation to the sites of infection in response to RV‐A16 and confirms our hypothesis. Affected are IFNα, IL‐1β, TNFα, IL‐6, IL‐8, CCL5 and IFNγ, all of which are involved in the activation processes of innate and/or adaptive immune cells [52, 53, 54, 55]. Such systemic defects in circulating immune cells could impair the local defence against infections in severe asthma. In consequence, RV‐A16 clearance might be delayed, which might contribute to exacerbations [8, 20]. The documented differences in the cell composition between the groups cannot explain the reduced IFNα‐, IL‐1β‐, IL‐6‐ and IL‐8‐responses to RV‐A16 in SA because all these cytokines are predominantly or exclusively produced by innate immune cells and rather not by lymphocytes [12, 18], which are reduced in the SA group. However, TNFα, CCL5 and IFNγ are also robustly produced in T‐cells [56] and the reduced CCL5 and IFNγ responses after 7 days could in part be explained by a reduced number of T‐cells in the PBMC fraction. TNFα, however, has not been induced by RV‐A16 after 7 days suggesting that its source is rather innate immune cells than T‐cells in the PBMC model because T‐cell activation might take longer than 24 h ex vivo. In line with our data, Pritchard and colleagues have demonstrated a decreased HRV‐induced IFNα production from PBMCs in mild‐to‐moderate atopic asthmatics compared to healthy individuals [57]. Similarly, cytokine responses of circulating T‐cells to bacterial infections are reduced in mild‐to‐moderate asthma and COPD, another obstructive lung disease, as well [38, 44]. However, in response to respiratory syncytial virus the cytokine responses of PBMCs are increased in COPD [52]. This indicates differences in molecular pathology of infection‐induced cytokine responses regarding the pathogen and/or type of obstructive lung disease. Airway epithelial cells from asthma patients with HRV infections have been reported to have a reduced capacity for Type I IFN synthesis compared to healthy individuals [19, 22]. In summary, it becomes clear that besides an overactivation of certain local immune cells like alveolar macrophages [37, 58], there is a reduced capacity in the production of cytokines required for infection defence in various circulating and local structural cells in direct response to many but not all pathogens in obstructive lung diseases. This might impair rapid pathogen clearance and contribute to exacerbations. Our data add the information that this also applies to the response of circulating immune cells to HRV or at least to the RV‐A16 strain in severe asthma.

T2‐high asthma is associated with high levels of blood eosinophils and FeNO. FeNO is increased in the two major phenotypes of T2 high asthma: eosinophilic and allergic asthma [59, 60]. Interestingly, IFNα production in response to RV‐A16 negatively correlated to FeNO but positively correlated to serum IgE, a sole marker of allergic asthma. This indicates that the defence against RV‐A16 might be more weakened in T2 high non‐allergic than in T2 high allergic asthma. CCL5 and IL‐6 responses to RV‐A16 also negatively correlated to FeNO. Both cytokines are involved in the recruitment of immune cells in response to infections [61, 62]. Thus, we carefully speculate that immune cell recruitment in response to RV‐A16 might particularly be impaired in T2 high asthma phenotypes. On the other hand, FeNO positively correlated to RV‐A16‐induced IL‐1β, and blood eosinophils positively correlated to IFNγ. Both cytokines are involved in the response of macrophages to viral infections [63, 64] suggesting that macrophage responses to RV‐A16 might be particularly impaired in T2 low asthma. Supportive, RV‐A16‐induced IFNγ also positively correlated with serum IL‐33, a marker that is particularly increased in T2 high including eosinophilic asthma [65]. However, it should be noted that most of the correlations are rather weak so that the interpretation of these data should be viewed with caution.

The cytokine responses to RV‐A16 could be induced in consequence of viral replication or by sensing incoming viral particles. In the PBMC fraction, the major RV‐A16 entry receptor ICAM‐1 is primarily expressed on monocytes [66] but also on dendritic cells [67], and at low levels constitutively on T‐, B‐ and NK‐cells [68, 69, 70]. This matches our data showing that RV‐A16 can replicate in PBMCs. Moreover, we showed that replication‐competent RV‐A16 induced all cytokines investigated in this study, except CCL2, much stronger than UV‐inactivated RV‐A16. This demonstrates that consequences of viral replication are the major trigger for cytokine expression in response to RV‐A16. However, CCL2 might be regulated primarily by direct recognition of viral particles or sensing of incoming vRNA after endocytosis.

This study has some limitations that should be considered. Firstly, we cannot address the reduced cytokine response in SA to specific cell types or specific cell–cell interactions of the PBMC fraction. Secondly, HRV infection is most robust at 33°C–35°C in the nasal epithelium [71, 72]. However, PBMCs cannot be cultured at 35°C and lower because this affects cell viability [73] and is not physiologically relevant. So, we used PBMC cultures at 37°C and were able to demonstrate that RV‐A16 replicates in and induces cytokine release from PBMCs under these conditions that might be suboptimal for RV‐A16 but are nonetheless physiologically and clinically relevant. Thirdly, our data do not provide information about the reason for the reduced activity of the immune cells in response to RV‐A16 in SA. It might be caused by differences in the efficiency of RV‐A16 attachment, entry, or replication mechanisms to/in specific cells of the PBMC fraction or by a reduced activity of related PRR pathways due to exhaustion caused by the abnormal inflammatory background in severe asthma. Bronchial epithelial cells have increased RV‐A16 replication and higher RV‐A16 loads in asthma compared to healthy subjects [22] but for immune cells, it is unclear. Finally, of the different HRV strains, HRV‐C has recently been recognized as the major trigger of asthma exacerbations in adults and children, although other strains, particularly HRV‐A strains, are also relevant [74, 75]. However, in contrast to children, disease severity, clinical characteristics, lung function data, serum inflammatory markers, hospitalization frequencies and systemic steroid use might not be different between adult patients positive for HRV‐C or other HRV species [74, 76]. This suggests that our data with RV‐A16 have clinical relevance for severe asthma in adults.

In conclusion, our data suggest that defects in circulating immune cells might cause a reduced cytokine response to RV‐A16 in severe asthma and, therefore, contribute to the increased susceptibility to HRV infections and to exacerbations. Depending on the cytokine, this molecular pathology might particularly apply to T2‐high asthma. However, our data also provide the first indication of impaired production of macrophage activating cytokines in response to RV‐A16 particularly in T2‐low severe asthma. Therefore, circulating immune cells might be considered as drug targets to reduce exacerbation rates in severe asthma.

## Author Contributions

**Kaschin Jamal Jameel:** investigation, formal analysis, visualization, writing – original draft, data curation, validation, methodology. **Sarah D. Yanik:** formal analysis, investigation, methodology. **Paul Bürger:** resources. **Kai Robin Pfeifer:** investigation, writing – review and editing. **Jürgen Knobloch:** conceptualization, formal analysis, supervision, funding acquisition, project administration, writing – review and editing, methodology, validation. **Simon Rohde:** resources. **Eike Bülthoff:** resources. **Daniel Weidinger:** investigation. **Juliane Kronsbein:** resources, project administration, methodology. **Birte Struck:** resources. **Matthias Tenbusch:** conceptualization, writing – review and editing, funding acquisition.

## Funding

This work was supported by the FoRUM program of the Ruhr‐University of Bochum (F801‐2014; to JKn and MT). SDY was supported by a short‐term research training fellowship of the European Respiratory Society (ERS). DW was funded by the Heinrich and Alma Vogelsang Stiftung. We acknowledge support by the open access publication funds of the Ruhr‐University Bochum. All funders were not involved in the study design, in the collection, analysis and interpretation of the data, in the writing of the report or in the decision to submit the paper for publication.

## Ethics Statement

This study was carried out according to the Code of Ethics of the World Medical Association. This study was approved by the Ethics Committee of the Ruhr‐ University Bochum, Germany (4257‐12, 4772‐13).

## Consent

All study subjects signed their informed consent for the use of their samples.

## Conflicts of Interest

J.K. has received research grants, speaker fees, and fees for participation on an advisory board from AstraZeneca, has received research grants from Sanofi, and has received speaker fees from GSK. All other authors declare no conflicts of interest.

## Data Availability

The data that support the findings of this study are available from the corresponding author upon reasonable request.

## References

[1] H. Backman , S. A. Jansson , C. Stridsman , et al., “Severe Asthma‐A Population Study Perspective,” Clinical and Experimental Allergy 49, no. 6 (2019): 819–828, 10.1111/cea.13378. 30817038

[2] K. F. Chung , S. E. Wenzel , J. L. Brozek , et al., “International ERS/ATS Guidelines on Definition, Evaluation and Treatment of Severe Asthma,” European Respiratory Journal 43, no. 2 (2014): 343–373, 10.1183/09031936.00202013. 24337046

[3] T. L. Jones , D. M. Neville , and A. J. Chauhan , “Diagnosis and Treatment of Severe Asthma: A Phenotype‐Based Approach,” Clinical Medicine (London, England) 18, no. Suppl 2 (2018): s36–s40, 10.7861/clinmedicine.18-2-s36. PMC6334025 29700091

[4] G. Kardas , P. Kuna , and M. Panek , “Biological Therapies of Severe Asthma and Their Possible Effects on Airway Remodeling,” Frontiers in Immunology 11 (2020): 1134, 10.3389/fimmu.2020.01134. PMC7314989 32625205

[5] K. S. Tan , H. H. Ong , Y. Yan , et al., “In Vitro Model of Fully Differentiated Human Nasal Epithelial Cells Infected With Rhinovirus Reveals Epithelium‐Initiated Immune Responses,” Journal of Infectious Diseases 217, no. 6 (2018): 906–915, 10.1093/infdis/jix640. 29228279

[6] A. Côté , K. Godbout , and L. P. Boulet , “The Management of Severe Asthma in 2020,” Biochemical Pharmacology 179 (2020): 114112, 10.1016/j.bcp.2020.114112. 32598948

[7] I. Mikhail and M. H. Grayson , “Asthma and Viral Infections: An Intricate Relationship,” Annals of Allergy, Asthma, and Immunology 123, no. 4 (2019): 352–358, 10.1016/j.anai.2019.06.020. PMC7111180 31276807

[8] T. Saraya , D. Kurai , H. Ishii , et al., “Epidemiology of Virus‐Induced Asthma Exacerbations: With Special Reference to the Role of Human Rhinovirus,” Frontiers in Microbiology 5 (2014): 226, 10.3389/fmicb.2014.00226. PMC4033317 24904541

[9] C. Lombardi , F. Gani , A. Berti , P. Comberiati , D. Peroni , and M. Cottini , “Asthma and COVID‐19: A Dangerous Liaison?,” Asthma Research and Practice 7, no. 1 (2021): 9, 10.1186/s40733-021-00075-z. PMC8279806 34261543

[10] K. Iikura , T. Katsunuma , S. Saika , et al., “Peripheral Blood Mononuclear Cells From Patients With Bronchial Asthma Show Impaired Innate Immune Responses to Rhinovirus In Vitro,” International Archives of Allergy and Immunology 155, no. Suppl 1 (2011): 27–33, 10.1159/000327262. 21646792

[11] M. L. Carroll , S. T. Yerkovich , A. L. Pritchard , J. M. Davies , and J. W. Upham , “Adaptive Immunity to Rhinoviruses: Sex and Age Matter,” Respiratory Research 11, no. 1 (2010): 184, 10.1186/1465-9921-11-184. PMC3024249 21194432

[12] S. Makris and S. Johnston , “Recent Advances in Understanding Rhinovirus Immunity,” F1000Research 7 (2018): F1000 Faculty Rev‐1537, 10.12688/f1000research.15337.1. PMC6173106 30345002

[13] Z. Yang , H. Mitländer , T. Vuorinen , and S. Finotto , “Mechanism of Rhinovirus Immunity and Asthma,” Frontiers in Immunology 12 (2021): 731846, 10.3389/fimmu.2021.731846. PMC8526928 34691038

[14] R. Channappanavar , A. R. Fehr , R. Vijay , et al., “Dysregulated Type I Interferon and Inflammatory Monocyte–Macrophage Responses Cause Lethal Pneumonia in SARS‐CoV‐Infected Mice,” Cell Host & Microbe 19, no. 2 (2016): 181–193, 10.1016/j.chom.2016.01.007. PMC4752723 26867177

[15] K. Chen and J. K. Kolls , “T Cell‐Mediated Host Immune Defenses in the Lung,” Annual Review of Immunology 31 (2013): 605–633, 10.1146/annurev-immunol-032712-100019. PMC3912562 23516986

[16] J. A. Acosta Davila and A. Hernandez De Los Rios , “An Overview of Peripheral Blood Mononuclear Cells as a Model for Immunological Research of Toxoplasma Gondii and Other Apicomplexan Parasites,” Frontiers in Cellular and Infection Microbiology 9 (2019): 24, 10.3389/fcimb.2019.00024. PMC6376612 30800644

[17] B. Allard , H. Levardon , P. Esteves , et al., “Asthmatic Bronchial Smooth Muscle Increases CCL5‐Dependent Monocyte Migration in Response to Rhinovirus‐Infected Epithelium,” Frontiers in Immunology 10 (2020): 2998, 10.3389/fimmu.2019.02998. PMC6956660 31969885

[18] P. W. Finn and T. D. Bigby , “Innate Immunity and Asthma,” Proceedings of the American Thoracic Society 6, no. 3 (2009): 260–265, 10.1513/pats.200807-064RM. PMC2677400 19387027

[19] M. Contoli , S. D. Message , V. Laza‐Stanca , et al., “Role of Deficient Type III Interferon‐Lambda Production in Asthma Exacerbations,” Nature Medicine 12, no. 9 (2006): 1023–1026, 10.1038/nm1462. 16906156

[20] D. R. Nagarkar , E. R. Bowman , D. Schneider , et al., “Rhinovirus Infection of Allergen‐Sensitized and ‐Challenged Mice Induces Eotaxin Release From Functionally Polarized Macrophages,” Journal of Immunology 185, no. 4 (2010): 2525–2535, 10.4049/jimmunol.1000286. PMC3208235 20644177

[21] D. Proud , “Role of Rhinovirus Infections in Asthma,” Asian Pacific Journal of Allergy and Immunology 29, no. 3 (2011): 201–208. 22053589

[22] P. A. Wark , S. L. Johnston , F. Bucchieri , et al., “Asthmatic Bronchial Epithelial Cells Have a Deficient Innate Immune Response to Infection With Rhinovirus,” Journal of Experimental Medicine 201, no. 6 (2005): 937–947, 10.1084/jem.20041901. PMC2213100 15781584

[23] H. Wangberg and K. Woessner , “Choice of Biologics in Asthma Endotypes,” Current Opinion in Allergy and Clinical Immunology 21, no. 1 (2021): 79–85, 10.1097/ACI.0000000000000708. 33306486

[24] T. Boonpiyathad , Z. C. Sözener , P. Satitsuksanoa , and C. A. Akdis , “Immunologic Mechanisms in Asthma,” Seminars in Immunology 46 (2019): 101333, 10.1016/j.smim.2019.101333. 31703832

[25] T. S. C. Hinks , S. J. Levine , and G. G. Brusselle , “Treatment Options in Type‐2 Low Asthma,” European Respiratory Journal 57, no. 1 (2021): 2000528, 10.1183/13993003.00528-2020. PMC7116624 32586877

[26] T. Kawakami and U. Blank , “From IgE to Omalizumab,” Journal of Immunology 197, no. 11 (2016): 4187–4192, 10.4049/jimmunol.1601476. PMC5123831 27864548

[27] F. C. Albers , C. Licskai , P. Chanez , et al., “Baseline Blood Eosinophil Count as a Predictor of Treatment Response to the Licensed Dose of Mepolizumab in Severe Eosinophilic Asthma,” Respiratory Medicine 159 (2019): 105806, 10.1016/j.rmed.2019.105806. 31751853

[28] H. Nagase , S. Ueki , and S. Fujieda , “The Roles of IL‐5 and Anti‐IL‐5 Treatment in Eosinophilic Diseases: Asthma, Eosinophilic Granulomatosis With Polyangiitis, and Eosinophilic Chronic Rhinosinusitis,” Allergology International 69, no. 2 (2020): 178–186, 10.1016/j.alit.2020.02.002. 32139163

[29] A. Badar , A. M. Salem , A. O. Bamosa , H. O. Qutub , R. K. Gupta , and I. A. Siddiqui , “Association Between FeNO, Total Blood IgE, Peripheral Blood Eosinophil and Inflammatory Cytokines in Partly Controlled Asthma,” Journal of Asthma & Allergy 13 (2020): 533–543, 10.2147/JAA.S274022. PMC7605921 33149625

[30] R. A. Dweik , P. B. Boggs , S. C. Erzurum , et al., “An Official ATS Clinical Practice Guideline: Interpretation of Exhaled Nitric Oxide Levels (FENO) for Clinical Applications,” American Journal of Respiratory and Critical Care Medicine 184, no. 5 (2011): 602–615, 10.1164/rccm.9120-11ST. PMC4408724 21885636

[31] D. B. Price , S. Bosnic‐Anticevich , I. D. Pavord , et al., “Association of Elevated Fractional Exhaled Nitric Oxide Concentration and Blood Eosinophil Count With Severe Asthma Exacerbations,” Clinical and Translational Allergy 9 (2019): 41, 10.1186/s13601-019-0282-7. PMC6702739 31452870

[32] S. Katoh , M. Ikeda , R. Shirai , et al., “Biomarkers for Differentiation of Patients With Asthma and Chronic Obstructive Pulmonary Disease,” Journal of Asthma 55, no. 10 (2018): 1052–1058, 10.1080/02770903.2017.1391281. 29035604

[33] R. Zhao , Y. Shi , N. Liu , and B. Li , “Elevated Levels of Interleukin‐33 Are Associated With Asthma: A Meta‐Analysis,” Immunity, Inflammation and Disease 11, no. 4 (2023): e842, 10.1002/iid3.842. PMC10116908 37102668

[34] H. Ganjian , C. Rajput , M. Elzoheiry , and U. Sajjan , “Rhinovirus and Innate Immune Function of Airway Epithelium,” Frontiers in Cellular and Infection Microbiology 10 (2020): 277, 10.3389/fcimb.2020.00277. PMC7316886 32637363

[35] N. Bedke , H. M. Haitchi , M. Xatzipsalti , S. T. Holgate , and D. E. Davies , “Contribution of Bronchial Fibroblasts to the Antiviral Response in Asthma,” Journal of Immunology 182, no. 6 (2009): 3660–3667. 10.4049/jimmunol.0802471 19265144

[36] K. Jamal Jameel , W. J. Gallert , S. D. Yanik , et al., “Biomarkers for Comorbidities Modulate the Activity of T‐Cells in COPD,” International Journal of Molecular Sciences 22, no. 13 (2021): 7187, 10.3390/ijms22137187. PMC8269158 34281240

[37] J. Knobloch , H. Hag , D. Jungck , K. Urban , and A. Koch , “Resveratrol Impairs the Release of Steroid‐Resistant Cytokines From Bacterial Endotoxin‐Exposed Alveolar Macrophages in Chronic Obstructive Pulmonary Disease,” Basic & Clinical Pharmacology & Toxicology 109 (2011): 138–143. 10.1111/j.1742-7843.2011.00707.x 21447053

[38] J. Knobloch , K. Schild , D. Jungck , et al., “The T‐Helper Cell Type 1 Immune Response to Gram‐Negative Bacterial Infections Is Impaired in COPD,” American Journal of Respiratory and Critical Care Medicine 183, no. 2 (2011): 204–214, 10.1164/rccm.201002-0199OC. 20709824

[39] J. R. Castillo , S. P. Peters , and W. W. Busse , “Asthma Exacerbations: Pathogenesis, Prevention, and Treatment,” Journal of Allergy and Clinical Immunology. In Practice 5, no. 4 (2017): 918–927, 10.1016/j.jaip.2017.05.001. PMC5950727 28689842

[40] A. Gillissen and M. Paparoupa , “Inflammation and Infections in Asthma,” Clinical Respiratory Journal 9, no. 3 (2015): 257–269, 10.1111/crj.12135. PMC7162380 24725460

[41] R. Hewitt , H. Farne , A. Ritchie , E. Luke , S. L. Johnston , and P. Mallia , “The Role of Viral Infections in Exacerbations of Chronic Obstructive Pulmonary Disease and Asthma,” Therapeutic Advances in Respiratory Disease 10 (2016): 158–174, 10.1177/1753465815618113. PMC5933560 26611907

[42] J. Knobloch , S. J. Chikosi , S. Yanik , J. Rupp , D. Jungck , and A. Koch , “A Systemic Defect in Toll‐Like Receptor 4 Signaling Increases Lipopolysaccharide‐Induced Suppression of IL‐2‐Dependent T‐Cell Proliferation in COPD,” American Journal of Physiology. Lung Cellular and Molecular Physiology 310, no. 1 (2016): L24–L39, 10.1152/ajplung.00367.2014. 26498252

[43] J. Knobloch , S. Panek , S. D. Yanik , et al., “The Monocyte‐Dependent Immune Response to Bacteria Is Suppressed in Smoking‐Induced COPD,” Journal of Molecular Medicine 97, no. 6 (2019): 817–828, 10.1007/s00109-019-01778-w. 30929031

[44] A. Koch , J. Knobloch , C. Dammhayn , et al., “Effect of Bacterial Endotoxin LPS on Expression of INF‐Gamma and IL‐5 in T‐Lymphocytes From Asthmatics,” Clinical Immunology 125, no. 2 (2007): 194–204, 10.1016/j.clim.2007.07.012. 17884733

[45] D. Dimitrova , V. Youroukova , E. Ivanova‐Todorova , K. Tumangelova‐Yuzeir , and T. Velikova , “Serum Levels of IL‐5, IL‐6, IL‐8, IL‐13 and IL‐17A in Pre‐Defined Groups of Adult Patients With Moderate and Severe Bronchial Asthma,” Respiratory Medicine 154 (2019): 144–154, 10.1016/j.rmed.2019.06.024. 31260861

[46] X. G. Jiang , X. D. Yang , Z. Lv , and P. H. Zhuang , “Elevated Serum Levels of TNF‐α, IL‐8, and ECP Can Be Involved in the Development and Progression of Bronchial Asthma,” Journal of Asthma 55, no. 2 (2018): 111–118, 10.1080/02770903.2017.1318141. 28399677

[47] M. Silvestri , M. Bontempelli , M. Giacomelli , et al., “High Serum Levels of Tumour Necrosis Factor‐Alpha and Interleukin‐8 in Severe Asthma: Markers of Systemic Inflammation?,” Clinical and Experimental Allergy 36, no. 11 (2006): 1373–1381, 10.1111/j.1365-2222.2006.02502.x. 17083347

[48] P. Davoodi , P. A. Mahesh , A. D. Holla , et al., “Serum Levels of Interleukin‐13 and Interferon‐Gamma From Adult Patients With Asthma in Mysore,” Cytokine 60, no. 2 (2012): 431–437, 10.1016/j.cyto.2012.05.012. 22698804

[49] D. S. Simpson , J. Pang , A. Weir , et al., “Interferon‐γ Primes Macrophages for Pathogen Ligand‐Induced Killing via a Caspase‐8 and Mitochondrial Cell Death Pathway,” Immunity 55, no. 3 (2022): 423–441, 10.1016/j.immuni.2022.01.003. PMC8822620 35139355

[50] I. Mahmutovic Persson , M. Menzel , S. Ramu , S. Cerps , H. Akbarshahi , and L. Uller , “IL‐1β Mediates Lung Neutrophilia and IL‐33 Expression in a Mouse Model of Viral‐Induced Asthma Exacerbation,” Respiratory Research 19, no. 1 (2018): 16, 10.1186/s12931-018-0725-z. PMC5781288 29361942

[51] A. L. Pritchard , O. J. White , J. G. Burel , M. L. Carroll , S. Phipps , and J. W. Upham , “Asthma Is Associated With Multiple Alterations in Anti‐Viral Innate Signalling Pathways,” PLoS One 9, no. 9 (2014): e106501, 10.1371/journal.pone.0106501. PMC4159236 25203745

[52] Y. Shi , C. H. Liu , A. I. Roberts , et al., “Granulocyte‐Macrophage Colony‐Stimulating Factor (GM‐CSF) and T‐Cell Responses: What We Do and Don't Know,” Cell Research 16, no. 2 (2006): 126–133, 10.1038/sj.cr.7310017. 16474424

[53] S. D. Yanik , K. Jamal Jameel , S. Rohde , et al., “Cytokine Production of Mononuclear Leukocytes in Response to Respiratory Syncytial Virus Is Increased in COPD but Suppressed in Non‐COPD Tobacco Smokers,” Molecular Medicine 31, no. 1 (2025): 237, 10.1186/s10020-025-01277-4. PMC12160369 40506734

[54] I. Ushach and A. Zlotnik , “Biological Role of Granulocyte Macrophage Colony‐Stimulating Factor (GM‐CSF) and Macrophage Colony‐Stimulating Factor (M‐CSF) on Cells of the Myeloid Lineage,” Journal of Leukocyte Biology 100, no. 3 (2016): 481–489, 10.1189/jlb.3RU0316-144R. PMC4982611 27354413

[55] H. Zelová and J. Hošek , “TNF‐α Signalling and Inflammation: Interactions Between Old Acquaintances,” Inflammation Research 62, no. 7 (2013): 641–651, 10.1007/s00011-013-0633-0. 23685857

[56] A. C. Hinrichs , S. L. M. Blokland , A. A. Kruize , F. P. J. Lafeber , H. L. Leavis , and J. A. G. van Roon , “CCL5 Release by CCR9+ CD8 T Cells: A Potential Contributor to Immunopathology of Primary Sjögren's Syndrome,” Frontiers in Immunology 1, no. 13 (2022): 887972, 10.3389/fimmu.2022.887972. PMC9198220 35720379

[57] A. L. Pritchard , O. J. White , J. G. Burel , and J. W. Upham , “Innate Interferons Inhibit Allergen and Microbial Specific T(H)2 Responses,” Immunology and Cell Biology 90, no. 10 (2012): 974–977, 10.1038/icb.2012.39. 22825591

[58] P. J. Barnes , “Alveolar Macrophages as Orchestrators of COPD,” COPD 1, no. 1 (2004): 59–70, 10.1081/COPD-120028701. 16997739

[59] M. Maniscalco , C. Candia , P. Ambrosino , M. G. Matera , and M. Cazzola , “Fractional Exhaled Nitric Oxide in Monitoring Biological Treatment for Severe Asthma in Adults: Clinical Implications and Future Perspectives,” Respiratory Medicine 12 (2026): 108647, 10.1016/j.rmed.2026.108647. 41534721

[60] N. Murugesan , D. Saxena , A. Dileep , M. Adrish , and N. A. Hanania , “Update on the Role of FeNO in Asthma Management,” Diagnostics (Basel) 13, no. 8 (2023): 1428, 10.3390/diagnostics13081428. PMC10137365 37189529

[61] W. G. Glass , H. F. Rosenberg , and P. M. Murphy , “Chemokine Regulation of Inflammation During Acute Viral Infection,” Current Opinion in Allergy and Clinical Immunology 3, no. 6 (2003): 467–473, 10.1097/00130832-200,312,000-00008. 14612671

[62] X. Li , C. Huang , K. R. Rai , and Q. Xu , “Innate Immune Role of IL‐6 in Influenza a Virus Pathogenesis,” Frontiers in Cellular and Infection Microbiology 15 (2025): 1605446, 10.3389/fcimb.2025.1605446. PMC12277322 40692679

[63] P. Jayaraman , I. Sada‐Ovalle , T. Nishimura , et al., “IL‐1β Promotes Antimicrobial Immunity in Macrophages by Regulating TNFR Signaling and Caspase‐3 Activation,” Journal of Immunology 190, no. 8 (2013): 4196–4204, 10.4049/jimmunol.1202688. PMC3622150 23487424

[64] J. L. Simpson , S. Phipps , K. J. Baines , K. M. Oreo , L. Gunawardhana , and P. G. Gibson , “Elevated Expression of the NLRP3 Inflammasome in Neutrophilic Asthma,” European Respiratory Journal 43, no. 4 (2014): 1067–1076, 10.1183/09031936.00105013. 24136334

[65] E. Gasiuniene , I. Janulaityte , Z. Zemeckiene , D. Barkauskiene , and B. Sitkauskiene , “Elevated Levels of Interleukin‐33 Are Associated With Allergic and Eosinophilic Asthma,” Scandinavian Journal of Immunology 89, no. 5 (2019): e12724, 10.1111/sji.12724. 30303258

[66] R. J. Stuyt , M. G. Netea , T. B. Geijtenbeek , B. J. Kullberg , C. A. Dinarello , and J. W. van der Meer , “Selective Regulation of Intercellular Adhesion Molecule‐1 Expression by Interleukin‐18 and Interleukin‐12 on Human Monocytes,” Immunology 110, no. 3 (2003): 329–334, 10.1046/j.1365-2567.2003.01747.x. PMC1783062 14632660

[67] D. N. Hart and T. C. Prickett , “Intercellular Adhesion Molecule‐2 (ICAM‐2) Expression on Human Dendritic Cells,” Cellular Immunology 148, no. 2 (1993): 447–454, 10.1006/cimm.1993.1126. 8098673

[68] M. L. Dustin , R. Rothlein , A. K. Bhan , C. A. Dinarello , and T. A. Springer , “Induction by IL 1 and Interferon‐Gamma: Tissue Distribution, Biochemistry, and Function of a Natural Adherence Molecule (ICAM‐1),” Journal of Immunology 137, no. 1 (1986): 245–254. 3086451

[69] M. R. Padros , M. I. Noli , and L. Fainboim , “Expression of ICAM‐1 (CD54) on Normal and Leukaemic B Cells: Implication for the Mixed Lymphocyte Reaction,” Clinical and Experimental Immunology 88, no. 2 (1992): 329–334, 10.1111/j.1365-2249.1992.tb03082.x. PMC1554302 1349273

[70] S. J. Lin and D. C. Yan , “ICAM‐1 (CD54) Expression on T Lymphocytes and Natural Killer Cells From Umbilical Cord Blood: Regulation With Interleukin‐12 and Interleukin‐15,” Cytokines, Cellular & Molecular Therapy 6, no. 4 (2000): 161–164, 10.1080/mccm.6.4.161.164. 11565953

[71] J. E. Gern , “The ABCs of Rhinoviruses, Wheezing, and Asthma,” Journal of Virology 84, no. 15 (2010): 7418–7426, 10.1128/JVI.02290-09. PMC2897627 20375160

[72] N. G. Papadopoulos , G. Sanderson , J. Hunter , and S. L. Johnston , “Rhinoviruses Replicate Effectively at Lower Airway Temperatures,” Journal of Medical Virology 58, no. 1 (1999): 100–104, 10.1002/(sici)1096-9071(199905)58:1<100::aid-jmv16>3.0.co;2-d. 10223554

[73] W. C. Olson , M. E. Smolkin , E. M. Farris , et al., “Shipping Blood to a Central Laboratory in Multicenter Clinical Trials: Effect of Ambient Temperature on Specimen Temperature, and Effects of Temperature on Mononuclear Cell Yield, Viability and Immunologic Function,” Journal of Translational Medicine 8, no. 9 (2011): 26, 10.1186/1479-5876-9-26. PMC3063218 21385453

[74] K. Kan‐O , Y. Washio , T. Fujimoto , et al., “Differences in the Spectrum of Respiratory Viruses and Detection of Human Rhinovirus C in Exacerbations of Adult Asthma and Chronic Obstructive Pulmonary Disease,” Respiratory Investigation 60, no. 1 (2022): 129–136, 10.1016/j.resinv.2021.08.009. 34580039

[75] J. Bizzintino , W. M. Lee , I. A. Laing , et al., “Association Between Human Rhinovirus C and Severity of Acute Asthma in Children,” European Respiratory Journal 37, no. 5 (2011): 1037–1042, 10.1183/09031936.00092410. PMC3024467 20693244

[76] Q. Xiao , S. Zheng , L. Zhou , et al., “Impact of Human Rhinovirus Types and Viral Load on the Severity of Illness in Hospitalized Children With Lower Respiratory Tract Infections,” Pediatric Infectious Disease Journal 34, no. 11 (2015): 1187–1192, 10.1097/INF.0000000000000879. 26267309

